# Structural analysis of a U-superfamily conotoxin containing a mini-granulin fold: Insights into key features that distinguish between the ICK and granulin folds

**DOI:** 10.1016/j.jbc.2024.107203

**Published:** 2024-03-18

**Authors:** Tiziano Raffaelli, David T. Wilson, Sebastien Dutertre, Julien Giribaldi, Irina Vetter, Samuel D. Robinson, Ashvriya Thapa, Antin Widi, Alex Loukas, Norelle L. Daly

**Affiliations:** 1 Australian Institute of Tropical Health and Medicine, James Cook University, Cairns, Australia; 2 IBMM, Univ Montpellier, CNRS, ENSCM, Montpellier, France; 3 Institute for Molecular Bioscience, The University of Queensland, Queensland, Australia; 4 School of Pharmacy, The University of Queensland, Queensland, Australia

**Keywords:** TxVIIB, conotoxin, disulfide-rich peptides, mini-granulin, inhibitor-cystine knot, AlphaFold

## Abstract

We are entering an exciting time in structural biology where artificial intelligence can be used to predict protein structures with greater accuracy than ever before. Extending this level of accuracy to the predictions of disulfide-rich peptide structures is likely to be more challenging, at least in the short term, given the tight packing of cysteine residues and the numerous ways that the disulfide bonds can potentially be linked. It has been previously shown in many cases that several disulfide bond connectivities can be accommodated by a single set of NMR-derived structural data without significant violations. Disulfide-rich peptides are prevalent throughout nature, and arguably the most well-known are those present in venoms from organisms such as cone snails. Here, we have determined the first three-dimensional structure and disulfide connectivity of a U-superfamily cone snail venom peptide, TxVIIB. TxVIIB has a VI/VII cysteine framework that is generally associated with an inhibitor cystine knot (ICK) fold; however, AlphaFold predicted that the peptide adopts a mini-granulin fold with a granulin disulfide connectivity. Our experimental studies using NMR spectroscopy and orthogonal protection of cysteine residues indicate that TxVIIB indeed adopts a mini-granulin fold but with the ICK disulfide connectivity. Our findings provide structural insight into the underlying features that govern formation of the mini-granulin fold rather than the ICK fold and will provide fundamental information for prediction algorithms, as the subtle complexity of disulfide isomers may be not adequately addressed by the current prediction algorithms.

Disulfide-rich peptides have attracted interest for the development of new therapeutics based on their potential to form stable structures and display potent and specific bioactivities (1). There has also been progress in using naturally occurring disulfide-rich peptides as scaffolds to engineer new bioactive molecules with enhanced stability (2, 3, 4, 5, 6). Understanding the oxidative folding of these peptides is critical to these engineering studies, and despite the knowledge gained on disulfide-rich peptide structures, and the recent advances in peptide and protein structure prediction (7), there is still much to learn regarding the folding of this class of peptide.

Cone snail venom peptides (conotoxins) represent a diverse source of disulfide-rich peptides (8). With more than 800 species of cone snails, there have been estimates of as many as 800,000 different conotoxins (9), which, to date, have been divided into 45 different gene superfamilies (10), and a variety of cysteine frameworks and connectivities. One of the most common structural motifs identified in conotoxins, and indeed more broadly in disulfide-rich peptides, is the inhibitor cystine knot (ICK). This motif, originally discovered in 1994 (11), comprises three disulfide bonds with the cysteine connectivity CysI-CysIV, CysII-CysV, and CysIII-CysVI, where two of the disulfide bonds form a ring through which the third bond threads.

Several studies have explored the evolution of the ICK fold (12, 13, 14), and structural links have been identified with the granulin fold (15, 16). The granulin framework is widespread throughout nature and contains a unique pattern of 12 cysteine residues, which form a laddered arrangement of six disulfide bonds and stacking of β-hairpins (17). A naturally occurring half-module also exists with six cysteine residues arranged in a CysI-CysIII, CysII-CysV, and CysIV-CysVI connectivity (18). The association of the ICK connectivity with the granulin structural motif was initially identified in conotoxin MiXXVIIA (G2-superfamily), where the peptide displayed similar structure to the N-terminal region of a human granulin module but contained three-disulfide bonds equivalent to the ICK connectivity (16). More recently, conotoxin Vc7.2 (H-superfamily) has also been shown to contain a granulin-like structure, despite containing the ICK disulfide connectivity, and the term “mini-granulin” structure was coined to describe this motif (15). Until the latter study, the cysteine framework VI/VII, present in Vc7.2, had been invariably associated with an ICK fold. There are several structurally uncharacterized conotoxins with the VI/VII cysteine framework with various inter-cysteine loop sizes and currently there is insufficient information available to predict the type of fold present.

To address this gap in knowledge, we determined the three-dimensional structure of a conotoxin containing the VI/VII cysteine framework purified from the venom of the molluscivorous snail *Conus textile*. We identified the precursor sequence based on analysis of available transcriptomic data and have referred to this peptide as TxVIIB to signify the unique precursor sequence and the presence in *C. textile*, but the mature peptide sequence is the same as Vc7.3 from *Conus victoriae* (19). Based on the precursor sequences, TxVIIB and Vc7.3 belong to the U-superfamily. The activities of these peptides are unknown, but a peptide with a very closely related mature peptide sequence, referred to as textile convulsant peptide (TCP), was reported from *C. textile* in 1992 and caused convulsions following intracranial injections in mice (20). Despite the TCP sequence being reported decades ago, no structural information is available on TCP or related sequences. Here, we show that conotoxin TxVIIB adopts a mini-granulin fold and that the inter-cysteine loop sizes appear to be playing a critical role in the formation of this motif. This information will assist in further development of artificial intelligence (AI) prediction of disulfide-rich peptide structures.

## Results

### Peptide extraction and isolation

**Figure 1:**
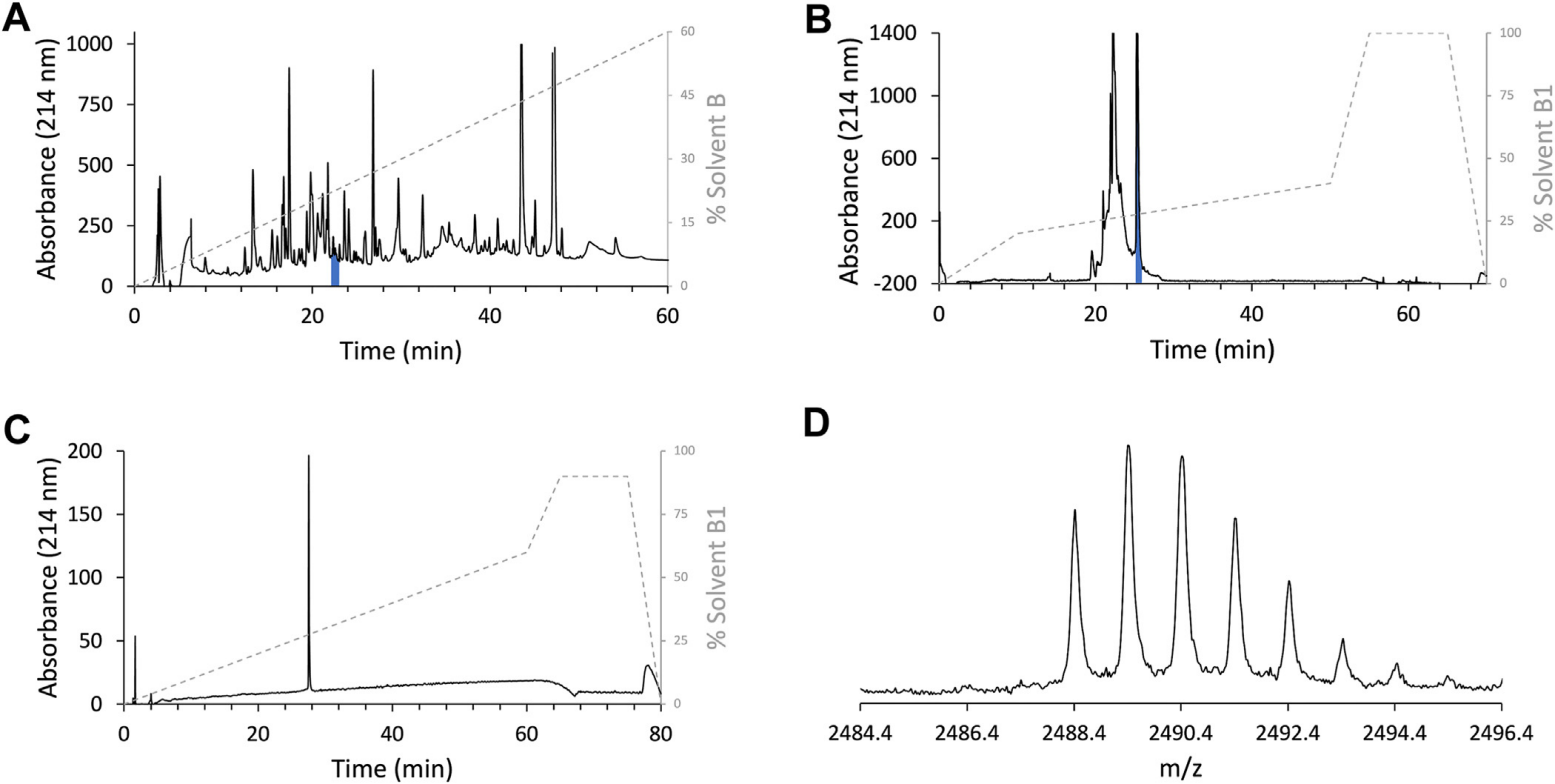
**TxVIIB identification and purification from *Conus textile* venom.***A*, preparative RP-HPLC of the whole venom. The peak highlighted in *blue* represents the fractions containing TxVIIB. *B*, semipreparative RP-HPLC of TxVIIB-containing fractions. The first eluted peak corresponds to a low molecular weight peptide and other compounds (shoulders). The sharp peak eluting second (in *blue*) corresponds to TxVIIB. *C*, analytical RP-HPLC trace of isolated TxVIIB used for NMR analysis and for coelution assay. *D*, SCIEX TOF/TOF 5800 MALDI MS spectrum of TxVIIB experimental mass [M + H]^+^ of *m/z* 2488.4 using CHCA matrix (theoretical [M + H]^+^ = 2488.9 *m/z*, error 200.9 ppm). CHCA, α-cyano-4-hydroxycinnamic acid; MS, mass spectrometry; RP-HPLC, reversed phase–high performance liquid chromatography.

Extracted *C. textile* venom was pooled prior to automated fractionation *via* reversed-phase high performance liquid chromatography (RP-HPLC). The chromatogram contained a large number of peaks (Fig. 1*A*). Fractions containing the mass corresponding to the peptide subsequently termed TxVIIB were identified by MALDI-TOF mass spectrometry (MS) analysis. The fractions containing the peptide of interest were combined (Fig. 1*B*) and purified a second time using a semipreparative column. The purity was assessed *via* analytical RP-HPLC and proved to be ≥ 95% (Fig. 1*C*). The experimental mass of TxVIIB, 2488.4 Da (theoretical mass 2488.9, error 200.9 ppm; Fig. 1*D*) corresponds closely to the mass of TCP as reported in ConoServer (21, 22), and this was our initial hypothesis for the peptide sequence. However, our analysis with NMR spectroscopy revealed some differences between the two peptides.

### Structural analysis with NMR spectroscopy

Analysis of the NMR spectra of the purified peptide, which included COSY, NOESY, TOCSY, and carbon and nitrogen heteronuclear single quantum coherence (HSQC) (Fig. S1), allowed unambiguous resonance assignments for the majority of the backbone amide groups and side chain protons. Most of the peaks in the [^1^H,^1^H]-TOCSY spectrum were sharp and well-resolved but some of the amide proton signals were overlapped, such as Cys^10^/Arg^21^, Ser^18^/Val^l6^, and Ala^13^/Cys^5^. Notably the alpha-proton of Val^6^ was significantly shielded compared to random coil values. The NOESY spectrum contained a large number of cross-peaks (Fig. S2), denoting a well-defined structure, and NOEs between Pro^11^ and Pro^12^ indicated proline 12 was in a *cis* conformation. All other proline residues appeared to be in a *trans* conformation based on sequential NOEs. The intercysteine Hβ-Hβ NOE data analysis revealed distinct peaks between Cys^5^/Cys^15^ and Cys^9^/Cys^20^ but showed some uncertainty regarding the remaining two cysteine residues (Cys^2^ and Cys^10^) due to spectral overlap. While this method has previously proven effective in elucidating disulfide bond connectivities in cysteine-rich peptides (23), it may yield misleading results in cases where all cysteine residues are closely packed, potentially due to the proximity of both nonbonded and bonded cysteine atoms (24).

The analysis of the spectra highlighted a difference with the TCP sequence (NCPYCVVYCCPPAYCEASGCRPP∗) as reported in ConoServer. Rather than a glutamic acid at position 16, our peptide contained a glutamine, and the C terminus was not amidated. These differences, based on the extra delta proton of glutamine in TOCSY and the absence of additional N at C terminus in [^1^H,^15^ N]-HSQC spectra were clear and result in the same mass as TCP but represent a distinct peptide we termed TxVIIB.

**Figure 2:**
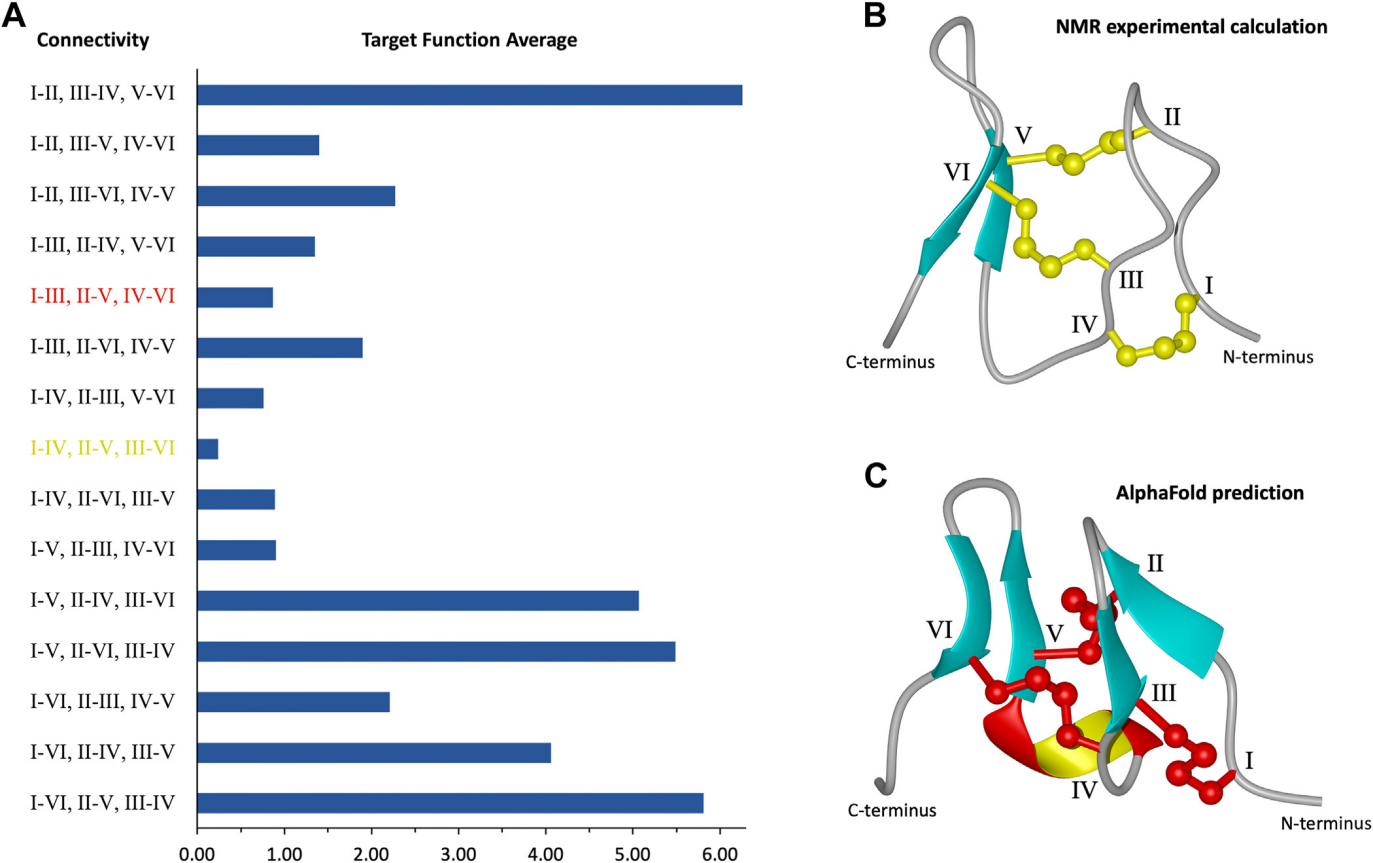
**TxVIIB disulfide bond connectivity analysis and three-dimensional structure.***A*, target functions, derived from CYANA (33), for the fifteen possible disulfide connectivities of TxVIIB are depicted. In *red*, the cysteine connectivity (granulin motif) corresponding to the AlphaFold (7) prediction is depicted. In *yellow*, the lowest target function with an ICK connectivity as calculated with NMR experimental data. *B,* TxVIIB (PDB code 80XR) three-dimensional structure with the disulfide bond connectivity corresponding to the lowest target function is shown. The cysteine residues are labeled using Roman numerals (I-VI), the disulfide bonds are colored in *yellow* and the β-sheets in *cyan*. The model shows a mini-granulin fold with an ICK connectivity. *C*, TxVIIB three-dimensional structure as predicted by AlphaFold. The cysteine residues are labeled using Roman numerals (I-VI), while the disulfide bonds are colored in *red*, the β-sheets in *cyan*, and α-helix in *red* and *yellow*. The structure shows a mini-granulin fold with a granulin connectivity. ICK, inhibitor cystine knot; PDB, Protein Data Bank.

TxVIIB has a mini-granulin fold consisting of a β-hairpin at the C terminus arranged in an antiparallel β-sheet conformation surrounded by three loops and turns stabilized into a compact structure by disulfide bonds between cysteine residues (Fig. 2*B*). The structure of TxVIIB stands out as unique when subjected to a VAST search (https://www.ncbi.nlm.nih.gov/Structure/VAST/vast.shtml). However, it is important to note that the core structure, represented by the β-hairpin and the connected two parallel disulfide bonds, has the same fold as H-Vc7.2 and the human granulin A N-terminal region. The superposition of TxVIIB 10 lowest energy conformations to human granulin A and H-Vc7.2 gives a RMSD of 0.631 Å and 0.369 Å, respectively, providing further evidence that the fold exhibited by TxVIIB is distinct from an ICK.

Calculation of the structures using CYANA of TxVIIB with the 15 possible disulfide connectivities indicated the connectivity often associated with framework VI/VII (Cys^I^-Cys^IV^, Cys^II^-Cys,^V^ and Cys^III^-Cys^VI^), where cysteine 2 is connected to cysteine 10, 5 to 15 and 9 to 20, had the lowest target function (Fig. 2*A*). The target function values are distributed within a range spanning from 0.24 (minimum) to 6.26 (maximum), with four connectivity combinations clustered around an approximate value of 0.85, such as Cys^I^-Cys^III^, Cys^II^-Cys^V^, Cys^IV^-Cys^VI^ (0.87), and Cys^I^-Cys^IV^, Cys^II^-Cys^VI^, Cys^III^-Cys^V^ (0.89).The target function for the connectivity patterns Cys^I^-Cys^III^, Cys^II^-Cys^V^, and Cys^IV^-Cys^VI^ associated with granulin folding and Cys^I^-Cys^IV^, Cys^II^-Cys^V^, and Cys^III^-Cys^VI^ associated with the ICK is only moderately different (the granulin fold is 3.5 times higher). However, upon imposing the granulin connectivity, six distance restraints violated. Most of the violations occur for restraints directly involved with the disulfide bridge between Cys^2^ and Cys^9^, but a NOE between Cys^2^ and Tyr^8^ also violates. Notably, the CYANA calculations also reveal a violation of the PSI angle restraint for the Cys^5^ residue. Only a limited number of experimental restraints violated with the ICK connectivity and highlights the challenge in discerning the native disulfide connectivity from NMR data alone. Overall, the majority of the disulfide combinations have a target function value exceeding the lowest figure by a factor of eight or more. The disulfide connectivity pattern identified is consistent with an ICK topology, as opposed to the distinctive Cys^I^-Cys^III^, Cys^II^-Cys^V^, and Cys^IV^-Cys^VI^ disulfide configuration associated with the mini-granulin fold observed in this peptide.

### Peptide synthesis and oxidation

**Figure 3:**
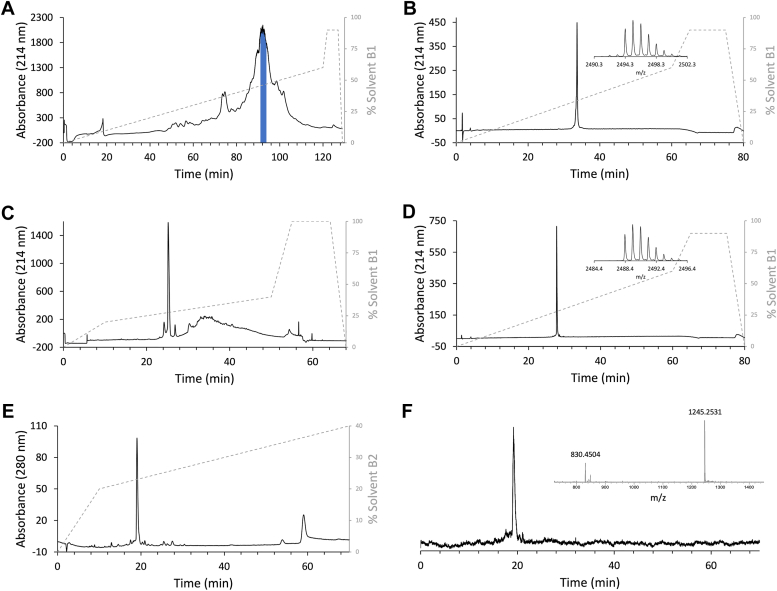
**RP-HPLC traces of randomly oxidized-TxVIIB purification steps, and coelution ESI-LCMS spectra.***A*, preparative RP-HPLC of the crude linear peptide. The main peak was fractionated, and the purest TxVIIB-containing fractions (in *blue*) were pooled for subsequent oxidation. *B*, analytical RP-HPLC and related MALDI MS spectrum of the selected TxVIIB-containing fraction. The inset provides evidence of cysteine reduction, with a [M + H]^+^ at *m/z* 2494.3 (theoretical [M + H]^+^ = 2494.9, error 240.5 ppm) that is six units higher than the nominal mass of native TxVIIB. *C*, semipreparative RP-HPLC after oxidation of combined TxVIIB fractions. The main peak corresponds to oxidized TxVIIB, and the broad bump to misfolded conformations of TxVIIB. *D*, analytical RP-HPLC of oxidized TxVIIB with a [M + H]^+^ at *m/z* 2488.4 (inset, theoretical [M + H]^+^ = 2488.9, error 200.9 ppm). *E*, ESI-LCMS absorbance trace of the coelution of native TxVIIB with the peptide produced by random oxidative folding (2:1 ratio). *F*, ESI-LCMS total ion chromatogram of coelution LC. *Inset*: ESI MS spectrum showing two TxVIIB positively charged ions at *m/z* 1245.2531 ([M + 2H]^2+^) and *m/z* 830.4504 ([M + 3H]^3+^), respectively (theoretical [M + 2H]^2+^ = 1245.4680, error 172.5456 ppm; and [M + 3H]^3+^ = 830.6453, error 234.6369 ppm). Gradients and columns used are described in Experimental procedures under Peptide purification and MS analysis. ESI-LCMS, electrospray ionization-LCMS; MS, mass spectrometry; RP-HPLC, reversed-phase high performance liquid chromatography.

Synthesis of TxVIIB was performed to validate that the proposed sequence and disulfide connectivity was identical to the native peptide. Randomly oxidized TxVIIB (roTxVIIB) was chemically synthesized using Fmoc-SPPS and oxidatively folded to form three disulfide bonds (as described in the Experimental procedures section). Random oxidation of the cysteine residues resulted in one main peak based on HPLC analysis, which was purified to give an yield of 5 to 7% from the reduced linear peptide (Fig. 3, *A*–*D*). The later eluting broad peaks are likely to correspond to misfolded peptide (different S-S connectivity(s)). The presence of misfolded peptide might be influenced by the presence of five proline residues (Pro3, Pro11, Pro12, Pro22, and Pro23) that are susceptible to *cis*/*trans* isomerization and the adjacent Cys^9^ and Cys^10^. The main synthetic isomer and native TxVIIB coeluted on LC/MS as shown in Figure 3, *E* and *F*. The chemical shifts derived from the NMR analysis were also consistent between the native and synthetic forms. These analyses indicated that the sequence and the disulfide connectivity were identical.

**Figure 4:**
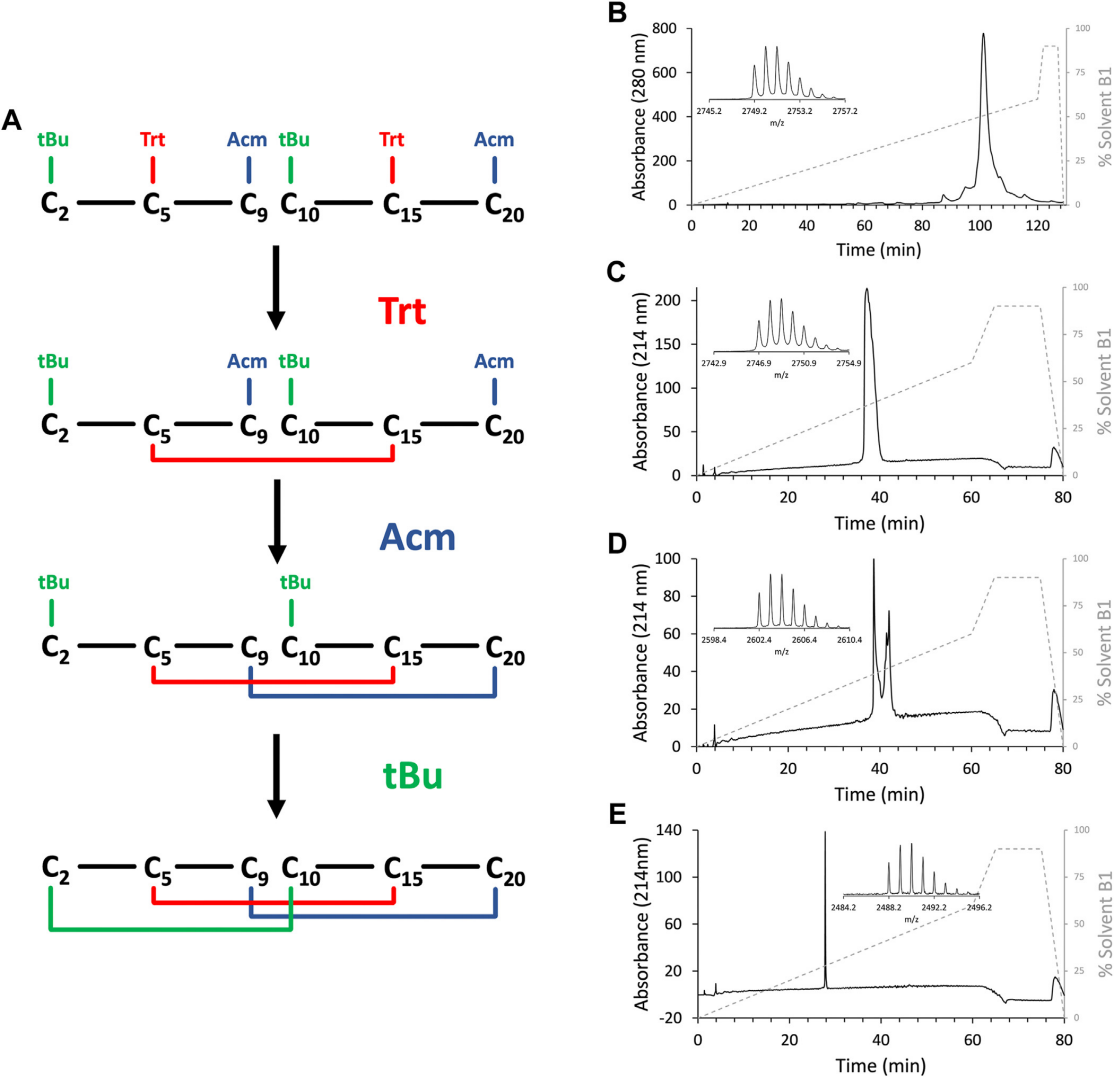
**TxVIIB stepwise oxidation strategy and RP-HPLC spectra of regioselective disulfide-bond formation.***A*, schematic procedure used for protected TxVIIB synthesis. *B*, preparative RP-HPLC of the reduced crude linear TxVIIB used for direct disulfide formation strategy. *Inset*: MS spectrum of the synthetic compound with the Acm and tBu protecting groups. [M + H]^+^ at *m/z* 2749.2 (theoretical [M + H]^+^ = 2749.3, error 36.4 ppm). *C*, analytical RP-HPLC of purified TxVIIB with the first disulfide bond, Cys^5^-Cys^15^. *Inset*: MS spectrum of the compound [M + H]^+^ at *m/z* 2746.9 (theoretical [M + H]^+^ = 2747.3, error 145.6 ppm). *D*, analytical RP-HPLC of purified TxVIIB with its first two disulfide bonds (oxidation of Cys^9^-Cys^20^). The two peaks represent the two isomers of this conformational stage. *Inset*: MS spectrum of the compound [M + H]^+^ at *m/z* 2602.4 (theoretical [M + H]^+^ = 2603.1, error 268.9 ppm). *E*, analytical RP-HPLC of purified TxVIIB with in its fully folded conformation (last disulfide bond Cys^2^-Cys^10^). Inset: MS spectrum of the compound [M + H]^+^ at *m/z* 2488.2 (theoretical [M + H]^+^ = 2488.9, error 281.2 ppm). MS, mass spectrometry; RP-HPLC, reversed-phase high performance liquid chromatography.

To chemically determine the disulfide bond connectivity present in TxVIIB, regioselective disulfide-bonds were formed using an orthogonal protecting-group strategy with trityl (Trt), acetamidomethyl (Acm), and *tert*-Butyl (tBu) for the respective pairs of cysteine residues according to the most likely disulfide connectivity from the NMR data; Trt for both Cys^5^ and Cys^15^, Acm for Cys^9^-Cys ^20^, and tBu to protect Cys^2^ and Cys^10^. The sequential order of disulfide formation was thus Cys^5^-Cys^15^, followed by Cys^9^-Cys^20^, and lastly Cys^2^-Cys^10^ (Fig. 4). The rationale for this order was based on previous studies on ICK peptides where the Cys^I^-Cys^IV^ bond appears to form last (25). Peptide was obtained in high purity and acceptable yield (∼5%) from the reduced linear peptide. Purification was performed after each deprotection/oxidation step, and HPLC analysis revealed the native peptide had the same elution time as stepwise oxidized TxVIIB (soTxVIIB). Intriguingly, more than one peak was evident following formation of the second disulfide bond. All of the peaks had the expected molecular weight and consequently appear to be conformational isomers, as subsequent formation of the final disulfide bond resulted in a single peak (Fig. 4*C*). Additionally, the two main peaks were purified and oxidized to independently form the final disulfide bond, and again formed the same single isomer (Fig. S3). NMR analysis indicated the synthetic version produced with regioselective formation of the disulfide bonds, soTxVIIB, and the native version of TxVIIB had the same structure based on chemical shift analysis and coelution by RP-HPLC (results not shown). A comparison of the [^1^H,^1^H]-TOCSY spectra and αH secondary-shifts for nTxVIIB, roTxVIIB, and soTxVIIB are given in Figs. S4 and S5, respectively, and showed perfect overlap and minimal deviation for the chemical shift of the three peptides.

### AlphaFold structure prediction

In order to further investigate the unexpected three-dimensional conformation of TxVIIB, we submitted an analysis to AlphaFold 2.0 (7). The five predicted models exhibited confidence scores ranging from 78.22 to 65.15 and notably, only one model showed a complete disulfide connectivity (Fig. S6*A*). Additionally, among the models with the highest confidence scores, it was observed that four shared the TxVIIB Cys^II^-Cys^V^ connectivity, with average figures for the local distance difference test of 68.90 and 80.25, respectively (Fig. S6*B*). The AlphaFold structure with complete disulfide connectivity, while similar to the experimental structure, displays more defined secondary structure. Despite the similarity between the predicted and experimental structures, the AI software predicted a different disulfide connectivity, specifically Cys^I^-Cys^III^, Cys^II^-Cys^V^, and Cys^IV^-Cys^VI^, which is consistent with the granulin connectivity pattern (Fig. 2*C*).

As expected, the AlphaFold structures predicted for H-Vc7.2 and MiXVVIIA closely matched the experimentally calculated structures as this data would have been available for the software training. Interestingly, the closely related peptide, TCP, was predicted to contain the ICK connectivity and the granulin fold, consistent with the result for TxVIIB rather than the experimentally predicted granulin disulfide connectivity (26) (Fig. S7).

### Bioactivity studies

TxVIIB was tested for bioactivity on a panel of voltage-gated ion channels on SH-SY5Y cells as described in the Experimental procedures. Although these channels are often targeted by toxins, the peptide displayed no discernible activity at concentrations up to a 30 μM (fluorescence response (area under the curve): Na_V_1.7: control 548.3 ± 17.4, TxVIIB 556.9 ± 11.7; Na_V_1.2: control 659.7 ± 31.7, TxVIIB 678.3 ± 21.0; α3 control 413.4 ± 4.7, TxVIIB 405.9 ± 5.5; α7 control 695.3 ± 53.3, TxVIIB 619.3 ± 34.6; Ca_V_2.2 control 792.5 ± 20.0, TxVIIB 692.7 ± 28.5; Ca_V_1.3 control 755.0 ± 14.8, TxVIIB 689.3 ± 29.5). TxVIIB also did not significantly inhibit Na_V_1.8 current using a state-dependent protocol as previously described (peak current TxVIIB (10 μM): 95.9 ± 1.8% of control). Similarly, when TxVIIB was administered at a concentration of 10 μM, it did not induce a direct increase in intracellular calcium levels in primary cultures of mouse dorsal root ganglion (DRG) neurons. In addition, TxVIIB was evaluated for its potential impact on cell proliferation due its structural similarity to granulins; however, no significant activity was observed.

## Discussion

Disulfide-rich frameworks and structural motifs are highly prevalent in nature, but the current study highlights that more experimental data are required to more accurately predict their three-dimensional structures. Links between the ICK and granulin cystine-rich modules are only just beginning to be recognized, and here we show that conotoxin TxVIIB is predicted to contain the granulin disulfide connectivity and structure based on AlphaFold, but experimental data indicate the ICK connectivity coupled with the granulin structure.

Analysis of the three-dimensional structure of TxVIIB indicates it lacks a hydrophobic core, suggesting that the surface features are primarily involved in stabilizing the structure. With only one positively charged residue, and no negatively charged residues, charge interactions do not appear to be involved in stabilizing the solution structure, but rather the surface exposed hydrophobic/aromatic residues might be involved. The reason for formation of the ICK disulfide connectivity, rather than the granulin connectivity predicted by AlphaFold is presumably related to formation of the correct arrangement of surface residues. However, we have shown here that structure calculations alone are insufficient to discern the important structural features as the structures calculated with the ICK and granulin connectivities have very similar overall structures and target functions. Mutational studies and structural analyses of related peptides are likely required to rationalize the formation of the mini-granulin fold with the ICK disulfide pattern in TxVIIB.

AI has become increasingly sophisticated in recent years and has been applied to a wide range of scientific fields, including biochemistry and protein structure prediction. AlphaFold is an AI-based protein prediction system that uses deep neural networks to predict the three-dimensional structures of proteins from their amino acid sequences. However, the accuracy of these predictions relies heavily on the quality and quantity of available experimental structural data. A comparison between the experimental structure of TxVIIB determined in our studies and a predicted model highlighted current limitations in AI structure prediction for small cysteine-rich peptides. While AI algorithms can use existing data to make predictions about the folding patterns of proteins, these predictions are only as accurate as the data they are based on. In cases where there are limited experimental data available, such as with disulfide-rich peptides, accuracy of AI predictions may be compromised. Conotoxins reveal a significant gap in structural knowledge, as only 205 out of the more than 8000 identified conotoxins have been structurally characterized to date (10). Moreover Alphafold, like many structure prediction algorithms, relies on evolutionary information derived from multiple sequence alignments to predict protein structures (7). The high sequence variability of cysteine-rich peptides, particularly outside of conserved cysteine residues, along with their limited conservation across evolutionary distant homologs, often result in suboptimal multiple sequence alignments. Consequently, AlphaFold may struggle to accurately infer the spatial arrangement of cysteine residues and their involvement in disulfide bond formation. Additionally, while AlphaFold integrates geometric constraints derived from known protein structures to guide predictions, these constraints may not fully capture the specific structural features of cysteine-rich peptides, such as disulfide bridges. This limitation can result in inaccuracies in the predicted structures. Recent discoveries, such as the ICK connectivity being associated with mini-granulin folding (15, 16), challenge even more conventional notions and underscore the dynamic nature of protein folding and structure determination. The present study highlights that the current limitations of AlphaFold in predicting the structures of cysteine-rich peptides can be attributed to the lack of sufficient training data, inadequate representation of evolutionary constraints, and challenges in capturing the geometric constraints associated with disulfide bond formation. Overcoming these limitations may require further refinement of the model architecture, incorporation of additional data sources, and development of specialized approaches tailored to the unique characteristics of cysteine-rich peptides.

Conotoxins that feature a cysteine framework VI/VII typically adopt an ICK fold, which is a highly stable globular structure formed by two disulfide bonds that create a ring perforated by a third disulfide bond. We have shown that TxVIIB, despite having the VI/VII cysteine framework and associated connectivity, folds with a granulin-like structure. This is likely due to the shorter inter-cysteine loop sizes in TxVIIB, which have only two to four amino acids in all of the inter-cysteine loops, compared to ICK-containing conotoxins, which have greater variation in the loop sizes (27). The experimentally determined connectivity for TxVIIB is in contrast to the pattern reported for the similar TCP, which was established solely through selective reduction and mass spectrometry experiments on a synthetic version of the peptide (26). The analysis of TCP might need to be revisited with additional approaches on native material to confirm if the two peptides do really differ in disulfide connectivity.

A search for sequences homologous to the peptide TxVIIB using blast to query the nr database revealed compelling results. A strong match was found with an E-value from 3e–19 to 4e–4, coupled with a remarkably high identity ranging from 100% to 91%, for 13 conotoxin sequences from eight different Conus species (*e.g. Conus ebraeus*, *Conus ammiralis*, and *Conus amadis*). These findings suggest that the uncommon structural feature characterized by the ICK disulfide connectivity combined with a granulin fold may be a prevalent structural motif.

The folding pathways associated with various ICK peptides have previously been studied, but there is limited information on the oxidative folding of granulin or mini-granulin peptides (25). The selective protection studies used herein indicate that the formation of the Cys^I^-Cys^IV^ bond as last oxidation step, allows formation of the native structure of TxVIIB. This is consistent with previous studies on EETI-II, a plant-derived cystine knot peptide (28) suggesting parallels in the folding of the two structure motifs. The current study also suggests that there are potentially two pathways available for formation of the native fold of TxVIIB, given that two peaks were present in the HPLC trace following formation of the second disulfide bond but both lead to the formation of the native fold. Based on the selective protection strategy used in these experiments it seems likely that these two peaks contain the same two disulfide bonds but are topological isomers, hence the difference in retention time (Fig. S3).

**Figure 5:**
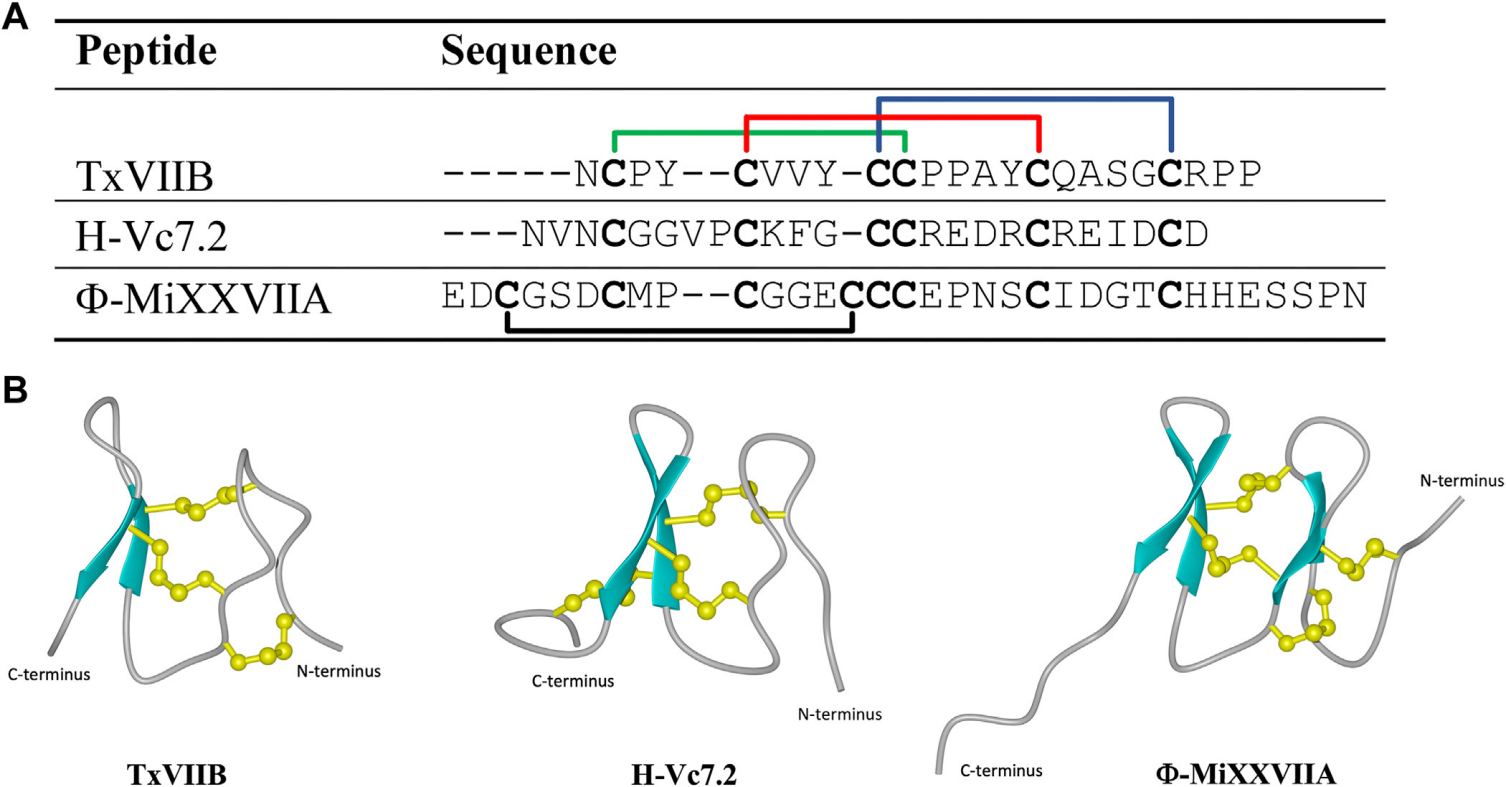
**Comparison of the three conotoxin structures containing a mini-granulin fold: TxVIIB (*Conus textile*), H-Vc7.2 (*Conus victoriae*), and Φ-MiXXVIIA (*Conus miles*).***A*, sequence alignment of the three peptides. The disulfide bond connections shared by the peptides are highlighted with colored lines (*green*, *red*, and *blue*). The additional disulfide bond of Φ-MiXXVIIA is represented with *black lines*. *B*, TxVIIB three-dimensional structure comparison with two different structural homologs. The structure of H-Vc7.2 (PDB code 6Q5Z) (15) is shown in the *middle*, while the structure of Φ-MiXXVIIA (PDB code 6PPC) (16) is shown on the *right*. PDB, Protein Data Bank.

**Figure 6:**
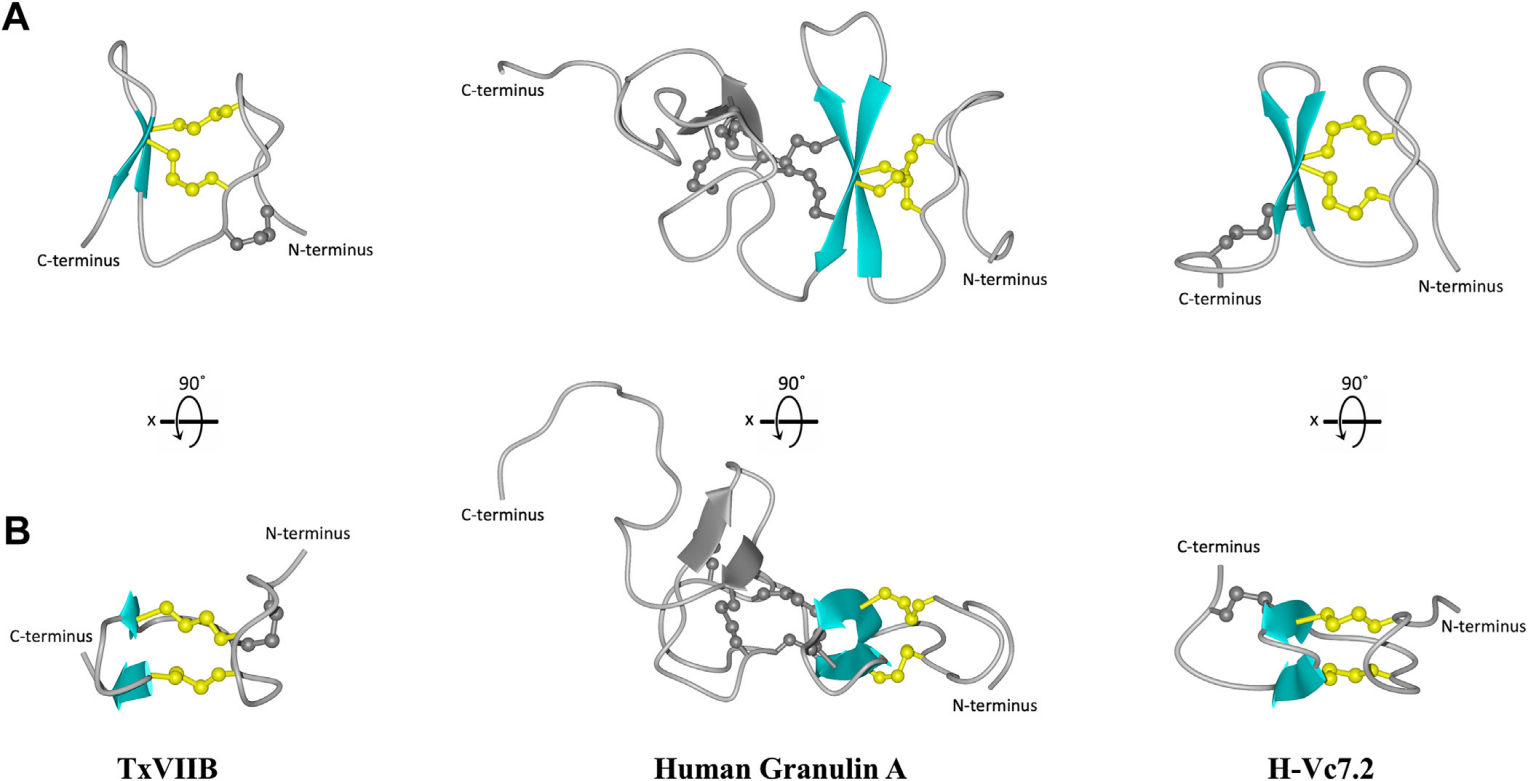
**Mini-granulin fold conserved core in TxVIIB, human granulin A, and H-Vc7.2.** The peptide structures (Human granulin A PDB code 2JYE) (37) are depicted from two distinct angles: the β-hairpin is shaded in *light blue*, the two parallel disulfide bonds are highlighted in *yellow*, and the nonconserved structures (additional β-sheets and disulfide bonds) are *grayed* out. The view shown in (*A*) is a 90° rotation along the *x*-axis with respect to the view shown in (*B*). PDB, Protein Data Bank.

Our identification of the mini-granulin fold in TxVIIB is consistent with other recent studies that have revealed conotoxins MiXXVIIA and H-Vc7.2 adopt the same fold (Fig. 5). The mini-granulin fold is defined as two stacked β-hairpins stabilized by two parallel disulfide bonds across a small β-sandwich (15). However, we propose a simplified description of this fold, characterized only by a single β-hairpin and two connected parallel disulfide bonds (Fig. 6). Indeed, the analysis of the 10 lowest energy structures for TxVIIB, H-Vc7.2, and the N-terminal region of human granulin A, revealed that 100%, 90%, and 60% of these structures, respectively, feature a single β-hairpin as the prominent structural element. To further investigate the important features that direct either ICK or mini-granulin folding in conotoxins, additional structural studies are necessary. Designing and synthesizing TxVIIB mutants with a higher number of residues in loop 1 and loop 2, as well as ICK-folding conotoxins with shorter sizes between the loops would allow for exploration of their folding patterns and biological activity, which still remains an open question. In fact, the functional implications of the mini-granulin folding pattern in conotoxins are not yet clear, as neither MiXXVIIA nor H-Vc7.2 has shown potent activity in a range of assays, and in the case of MiXXVIIA, only limited antiapoptotic and cell-proliferative activity have been reported.

To summarize, our study has demonstrated that conotoxin TxVIIB, which shares the same VI/VII cysteine framework as many ICK-folding conotoxins, exhibits a granulin-like folding pattern, most likely due to the shorter inter-cysteine loop sizes in TxVIIB. Our findings indicate that further research is necessary to fully explore the functional implications of the mini-granulin folding pattern in conotoxins and other peptides. Moreover, our results highlight the importance of experimental data in accurately predicting protein folding patterns and provides insight into the factors that influence peptide structure. With the discovery of the mini-granulin fold evident among another conotoxin superfamily, there is a clear need to characterize more conotoxin structures in order to better implement AI algorithms and gain a more complete understanding of the complex processes that govern protein folding.

## Experimental procedures

### Peptide extraction and isolation

Predatory venom from *C. textile* (collected from 5 to 10 specimens over one year) was collected as previously described (29). Lyophilized venom (5 mg) was resuspended in 600 μl of solvent A (H_2_O, 0.1% formic acid). The resuspended venom was centrifuged at 10,000*g* for 5 min to remove insoluble particles. The supernatant was injected into an HPLC UltiMate 3000 Standard LC system (Thermo Fisher Scientific), equipped with a Syncronis column C_18_ 250 × 4.6 mm; 5 μm (Thermo Fisher Scientific). The venom components were eluted using a linear 1%/min gradient of solvent B (Acetonitrile (MeCN), 0.1% formic acid) at a flow rate of 1 ml/min over 80 min. One min fractions were automatically collected into a 2 ml 96-well plate. Venom fractions containing TxVIIB were pooled and further purified using a semipreparative column (Phenomenex Aeris 5 μm, PEPTIDE XB-C18, 100 Å pore size, 250 mm × 10.0 mm) with a gradient of 0 to 20% solvent B1 in 10 min, 20 to 40% solvent B1 in 40 min and 40 to 100% solvent B1 in 5 min. The flow rate was 3 ml/min, peak detection was at 214 nm, solvent A1 was 0.05% TFA in water, and solvent B1 was 0.045% TFA, 10% water in MeCN. The purity of the peptide was assessed using analytical RP-HPLC on a C_18_ analytical column (Phenomenex Kinetex 3.5 μm, XB-C18, 150 × 4.6 mm), and peaks were detected at 214 nm. The gradient used was 0 to 60% solvent B1 in 60 min and 60 to 90% solvent B1 in 5 min at 1 ml/min. The TxVIIB venom fraction was freeze-dried and stored at −20 °C until use.

### Peptide synthesis and oxidation

TxVIIB (NCPYCVVYCCPPAYCQASGCRPP) was synthesized by manual solid-phase peptide synthesis (SPPS) using Fmoc SPPS chemistry. Peptides were assembled on 2-chlorotrityl chloride resin (Peptides International). Amino acids were activated using O-(1H-6- chlorobenzotriazol-1-yl)-1,1,3,3-tetramethyluronium hexafluorophosphate in peptide synthesis grade dimethylformamide, and Fmoc protecting groups were removed using 20% piperidine in dimethylformamide. Peptides were cleaved using a mixture of 95% TFA/2.5% triisopropyl silane/2.5% water at room temperature for 2.5 h. The TFA was removed by evaporation with nitrogen, and ice-cold diethyl ether was added to precipitate the peptides. Diethyl ether was then removed by filtration, peptides were dissolved in 45% MeCN/water containing 0.1% TFA and subsequently lyophilized and purified (purification method described in paragraph below). Two strategies were used for TxVIIB synthesis and oxidative folding: (i) random oxidation to generate roTxVIIB, and (ii) stepwise oxidation using three different cysteine protecting groups to produce soTxVIIB.

For the first strategy, all the cysteine residues were introduced with trityl protecting groups. roTxVIIB was resuspended in MeCN (2 mg/ml) and diluted in 100 mM ammonium bicarbonate (pH 8.3) for 24 h (2 h room temperature and 22 h at 4 °C) with a peptide concentration of 0.25 mg/ml to allow oxidation of cysteine residues. Monitoring of the oxidation was performed *via* HPLC using an Agilent 1260 Infinity HPLC system and MS using a SCIEX TOF/TOF 5800 MALDI (SCIEX).

For the second strategy, synthesis of soTxVIIB was performed using trityl (Trt-), acetamidomethyl (Acm-), and tert-butyl (tBu-) protected cysteine residues, according to the predicted disulfide connectivity (see NMR section). The order of disulfide bond formation was guided by preliminary experiments where a peptide was synthesized with the Cys^II^-Cys^V^ formed as the first bond, and the remaining two bonds were formed randomly. This approach enabled formation of the native fold (data not shown). Based on these experiments, the Cys^II^-Cys^V^ bond in soTxVIIB was formed first using Trt protecting groups, followed by Cys^III^-Cys^VI^ and then Cys^I^-Cys^IV^ which were protected with Acm and tBu groups respectively. The Trt group was removed during TFA cleavage from the resin; hence, the partially Acm- and tBu-protected peptide was purified, lyophilized, and subjected to a stepwise (steps I-III) deprotection/oxidation protocol (Fig. 4*A*). For the formation of the first disulfide bond (step I), the peptide was oxidized following the protocol used for roTxVIIB, then purified and freeze-dried. The lyophilized single-bond precursor peptide was used for the deprotection/oxidation (one pot reaction) of the Acm groups (step II) by dissolving the peptide in a mixture 94.8: 4.7: 0.5 (v/v/v) of ice cold 50% aq. acetic acid, 1 M ice cold HCl, and treated with 0.1 M iodine for a final peptide concentration of ∼1 mg/ml. The mixture was immediately flushed with nitrogen, and gently stirred for 1 h at 4 °C. The reaction was quenched by adding a 1 M solution of ascorbic acid until the solution was colorless and monitored *via* HPLC and MS until complete oxidation was confirmed. Following purification and lyophilization of the semiprotected peptide, the final deprotection/oxidation of Cys-tBu was performed (step III). Peptide (1 mg) was dissolved in 2 ml of a 97.9: 2: 0.1 (v/v/v) cocktail of TFA, dimethylsulfoxide, and anisole, then shaken for 24 h in the cold room (4 °C). The TFA was removed with nitrogen, the peptide precipitated with cold diethyl ether, and washed with 45% MeCN/water containing 0.1% TFA. The reaction was monitored *via* HPLC and MS to confirm the oxidation.

### Peptide purification and MS analysis

The crude linear peptides were purified with reversed-phase HPLC equipped with a preparative column (Phenomenex Jupiter 10 μm, C_18_, 300 Å pore size, 250 mm × 21.2 mm). The peptides were eluted with a linear gradient of 0 to 60% solvent B1 in 120 min and monitored at 214 and 280 nm at a flow rate of 5 ml/min. Synthetic TxVIIB partially and fully oxidized fractions were purified using RP-HPLC on a semipreparative column (Phenomenex Aeris 5 μm, PEPTIDE XB-C18, 100 Å pore size, 250 mm × 10.0 mm) with a flow rate of 3 ml/min. Fully oxidized roTxVIIB was purified with a linear gradient of 0.5%/min from 20% to 40% of solvent B1 over 40 min. The product of the three oxidation steps of soTxVIIB were purified with the sequent gradients: 0.5%/min of Solvent B1 in 120 min following oxidation I, 0.5%/min from 30% to 50% of Solvent B1 in 40 min after oxidation II, and 1%/min of Solvent B1 in 60 min after oxidation III. The purity and the oxidation of the peptides was assessed using analytical RP-HPLC on a C_18_ analytical column (Kinetex 3.5 μm, XB-C18, 150 × 4.6 mm), and peaks were detected at 214 nm. The gradient used was 0 to 60% solvent B1 in 60 min and 60 to 90% solvent B1 in 5 min at 1 ml/min. The masses of the products from the peptide synthesis and each deprotection/oxidation step were confirmed by MALDI-TOF MS. MALDI-MS samples were spotted on 384-well stainless-steel target plates using 0.75 μl of sample and 0.75 μl of α-cyano-4-hydroxycinnamic acid (Sigma-Aldrich) matrix at 7.5 mg/ml in 50% MeCN/0.1% TFA. Calibration was performed before spectra collection using SCIEX TOF/TOF calibration mix (PN#: 4333604; SCIEX). Spectra were acquired in linear and reflector positive ion mode from *m/z* 1000 to 10,000 and *m/z* 800 to 4500 and averaged over 2500 and 2000 laser shots, respectively. Coelution was performed on the native (nTxVIIB) and synthetic peptide (roTxVIIB) by liquid LC/MS using a Shimadzu LCMS-2020 mass spectrometer coupled to a Shimadzu Prominence HPLC system. A mixture of synthetic and native peptide at a ratio of 1:2 was injected *via* an autosampler (Shimadzu SIL-20ACHT) onto a RP-HPLC column (Phenomonex Aeris 150 mm × 2.1 mm 3.6 μm PEPTIDE XB-C18 100 Å) at 30 °C. Solvent (solvent A2: 0.1% formic acid/H_2_O; solvent B2: 90% MeCN/0.1% formic acid/H_2_O) was delivered *via* Shimadzu LC-20AD pumps at a flow rate of 0.250 ml/min. Samples were eluted with a gradient of 0.3% solvent B1, 20 to 40% in 60 min. The UV absorbance was monitored at 214 and 280 nm using a Shimadzu SPD-20A detector. Mass spectra were collected in positive ion mode over a scan range of *m/z* 250 − 2000 with a detector voltage of 1.55 kV, nebulizing gas flow of 1.5 L/min, and drying gas flow of 3.0 L/min. Data were collected and analyzed using the Shimadzu LabSolutions v5.96 software.

### NMR spectroscopy and structure determination

Lyophilized samples were dissolved in a solution of 500 μl water and 50 μl D_2_O for a final peptide concentration of 0.2 mM. NMR spectra were acquired at a temperature of 290 K using a 600 MHz Bruker Avance III spectrometer (Bruker) equipped with a cryoprobe. Chemical shifts were referenced to external sodium trimethylsilylpropanesulfonate. Two-dimensional spectra included [^1^H,^1^H]-TOCSY, [^1^H,^1^H]-NOESY, [^1^H,^1^H]-COSY, [^1^H,^13^ C]-HSQC, and [^1^H,^15^ N]-HSQC. TOCSY and NOESY mixing times of 80 ms and 200 ms, respectively, were used. All NMR spectra were processed and analyzed using TopSpin (Bruker), and peaks were assigned using Collaborative Computing Project for NMR analysis (CcpNMR) based on the approach described by Wüthrich (30, 31). Phi and psi backbone torsion-angle restraints obtained from ^1^H and ^13^C chemical-shift analyses were predicted using TALOS-N (32). In addition, the disulfide bond connectivity for every theoretically possible conformation (6 cysteines generate 15 different possible disulfide connectivities) was included in the structure calculations. Distance restraints were derived by automated [^1^H,^1^H]-NOESY spectra assignment, and the 20 structures with the lowest energy were calculated using the program CYANA (33). The three-dimensional structures were visualized using MOLMOL (34). The structural data have been deposited in the Protein Data Bank (PDB) (35) under the PDB code 8UXR, while the corresponding chemical shifts have been deposited in the Biological Magnetic Resonance Bank (http://www.bmrb.wisc.edu/ (36)) with the BMRB ID 31126.

### Ca^2+^ imaging assays

The human neuroblastoma cell line SH-SY5Y endogenously expressing Na_V_1.2, Na_V_1.7, Ca_V_2.2, Ca_V_1.3, as well as α7 and α3-containing nicotinic acetylcholine receptors (nAChRs) was cultured in RPMI media supplemented with L-glutamine and 15% fetal bovine serum and plated at a cell density of 15,000 cells/well in black-walled, clear bottom 384-well plates. Following 48 h in culture at 37 °C/5% CO_2_, cells were loaded with Calcium 4 No-wash dye (Molecular Devices) in physiological salt solution (composition in mM: NaCl 140, D-glucose 11.5, KCl 5.9, MgCl_2_ 1.4, NaH_2_PO_4_ 1.2, NaHCO_3_ 5, CaCl_2_ 1.8, and Hepes 10) for 30 min, and changes in fluorescence (excitation 470–495 nm; emission 515–575 nm) in response to addition of peptide (8-point serial dilution from 30–0.5 μM) were measured for 300 s using a FLIPR^Penta^ fluorescent plate reader (Molecular Devices). Responses mediated *via* endogenously expressed ion channels were subsequently elicited by addition of: choline (30 μM)/PNU-120596 (10 μM) for α7 nAChR-mediated responses; nicotine (30 μM) for α3β2/α3β4 nAChR-mediated responses; KCl (90 mM)/CaCl_2_ (5 mM) for Ca_V_1.3-mediated responses; KCl (90 mM)/CaCl_2_ (5 mM) + nifedipine (10 μM) for Ca_V_2.2-mediated responses; vertridine (70 μM) for Na_V_1.2-mediated responses; and veratridine (4 μM)/OD1 (300 nM) for Na_V_1.7-mediated responses. Raw fluorescence readings were converted to response over baseline using the analysis tool Screenworks 3.1.1.4 (Molecular Devices) and were expressed relative to the maximum increase in fluorescence of control responses and plotted using GraphPad Prism 9.3.1 (www.graphpad.com).

### Automated patch-clamp electrophysiology

CHO cells stably expressing hNa_V_1.8/β3 in a tetracycline-inducible system (ChanTest.) were grown in minimum essential medium media containing 10% fetal bovine serum and 2 mM L-glutamine. Cells were grown at 37 °C with 5% CO_2_ and passaged every 3 to 4 days using TrypLE Express (Thermo Fisher Scientific) at 70 to 80% confluency. To induce expression of hNa_V_1.8, cells were cultured in the presence of 1 μg/ml tetracycline for 48 h at 27 °C.

Automated whole-cell patch-clamp recordings were performed on QPatch-16 (Sophion Bioscience) using multihole (QPlate 16X with a standard resistance of 0.2 ± 0.04 MΩ). The extracellular solution contained (in mM): 145 NaCl, 4 KCl, 1 MgCl_2_, 2 CaCl_2_, 10 glucose, and 10 Hepes, adjusted to pH 7.4 with NaOH and osmolarity to 305 mOsm with sucrose. The intracellular solution consisted of (in mM): 140 CsF, 5 CsOH, 1 EGTA, 10 NaCl, and 10 Hepes, adjusted to pH 7.4 with CsOH and osmolarity to 320 mOsm with sucrose. Subsequently, 1 μM tetrodotoxin was added to the extracellular solution to inhibit endogenous tetrodotoxin-sensitive current in CHO cells. To assess the partially inactivated state of hNa_V_1.8 in the presence of 10 μM TCP, a 50-ms test pulse from −90 mV to 10 mV was applied after an 8000-ms conditioning pulse to −40 mV with a 20-ms recovery period.

### DRG assay

DRG cells were isolated from 8-week-old male C57BL/6 mice purchased from the Animal Resources Center. DRGs were dissociated, then cells plated in Dulbecco’s modified Eagle’s medium (Gibco) containing 10% fetal bovine serum (Assay matrix) and penicillin/streptomycin (Gibco) on a 96-well poly-D-lysine-coated culture plate (Corning) and maintained overnight. Cells were loaded with Fluo-4 AM calcium indicator, according to the manufacturer’s instructions (Thermo Fisher Scientific). After loading (1 h), the dye-containing solution was replaced with assay solution (Hanks’ balanced salt solution, 20 mM Hepes). Images were acquired at 10× objective at one frame/s (excitation 485 nm, emission 521 nm). Fluorescence corresponding to [Ca^2+^]_*i*_ of ∼150 cells per experiment was monitored in parallel using a Nikon Ti-E deconvolution inverted microscope, equipped with a Lumencor Spectra LED Light source. Baseline fluorescence was monitored for 30 s. At 30 s, assay solution was replaced with assay solution, then at 1 min with test peptide (in assay solution) and monitored for 2 min before being replaced with KCl (30 mM; positive control). Experiments were approved by the UQ AEC (approval TRI/IMB/093/17).

### The real time xCELLigence cell proliferation assay

The human skin normal fibroblast cell line, 1BR.3.GN, obtained from the European Collection of Authenticated Cell Cultures was confirmed to be *mycoplasma* free and cultured to test effects of TxVIIB on cell proliferation on a xCELLigence SP system (ACEA Bioscience) as described previously (18). A plant extract, which previously shown enhanced cell proliferation (unpublished results), was used as a positive control in the assay, serum-free media was used as a negative control. Day 4 cell proliferation rates were compared between treatment and control wells, and a one-way ANOVA test was used for multiple comparisons with Holm-Sidak’s correction, using GraphPad Prism 9.3.1.

## Data availability

Data are presented in the manuscript.

## Supporting information

This article contains supporting information (26, 38).

## Conflict of interest

The authors declare that they have no conflicts of interest with the contents of this article.

## References

[1] Craik D.J., Daly N.L., Waine C. (2001). The cystine knot motif in toxins and implications for drug design. Toxicon.

[2] Kintzing J.R., Cochran J.R. (2016). Engineered knottin peptides as diagnostics, therapeutics, and drug delivery vehicles. Curr. Opin. Chem. Biol..

[3] Craik D.J., Du J. (2017). Cyclotides as drug design scaffolds. Curr. Opin. Chem. Biol..

[4] Camarero J.A., Campbell M.J. (2019). The potential of the cyclotide scaffold for drug development. Biomedicines.

[5] Chaudhuri D., Aboye T., Camarero J.A. (2019). Using backbone-cyclized Cys-rich polypeptides as molecular scaffolds to target protein-protein interactions. Biochem. J..

[6] Poth A.G., Chan L.Y., Craik D.J. (2013). Cyclotides as grafting frameworks for protein engineering and drug design applications. Biopolymers.

[7] Jumper J., Evans R., Pritzel A., Green T., Figurnov M., Ronneberger O. (2021). Highly accurate protein structure prediction with AlphaFold. Nature.

[8] Nguyen L.T.T., Craik D.J., Kaas Q. (2023). Bibliometric review of the literature on cone snail peptide toxins from 2000 to 2022. Mar. Drugs.

[9] Himaya S.W.A., Lewis R.J. (2018). Venomics-accelerated cone snail venom peptide discovery. Int. J. Mol. Sci..

[10] Kaas Q., Westermann J.C., Craik D.J. (2010). Conopeptide characterization and classifications: an analysis using ConoServer. Toxicon.

[11] Pallaghy P.K., Nielsen K.J., Craik D.J., Norton R.S. (1994). A common structural motif incorporating a cystine knot and a triple-stranded beta-sheet in toxic and inhibitory polypeptides. Protein Sci..

[12] Smith J.J., Hill J.M., Little M.J., Nicholson G.M., King G.F., Alewood P.F. (2011). Unique scorpion toxin with a putative ancestral fold provides insight into evolution of the inhibitor cystine knot motif. Proc. Natl. Acad. Sci. U. S. A..

[13] Gao B., Harvey P.J., Craik D.J., Ronjat M., De Waard M., Zhu S. (2013). Functional evolution of scorpion venom peptides with an inhibitor cystine knot fold. Biosci. Rep..

[14] Zhu S., Darbon H., Dyason K., Verdonck F., Tytgat J. (2003). Evolutionary origin of inhibitor cystine knot peptides. FASEB J..

[15] Nielsen L.D., Foged M.M., Albert A., Bertelsen A.B., Soltoft C.L., Robinson S.D. (2019). The three-dimensional structure of an H-superfamily conotoxin reveals a granulin fold arising from a common ICK cysteine framework. J. Biol. Chem..

[16] Jin A.H., Dekan Z., Smout M.J., Wilson D., Dutertre S., Vetter I. (2017). Conotoxin Phi-MiXXVIIA from the superfamily G2 Employs a Novel cysteine framework that Mimics granulin and displays anti-apoptotic activity. Angew. Chem. Int. Ed Engl..

[17] Dastpeyman M., Smout M.J., Wilson D., Loukas A., Daly N.L. (2018). Folding of granulin domains. Pept. Sci.

[18] Takjoo R., Wilson D., Bansal P.S., Loukas A., Smout M.J., Daly N.L. (2020). Folding of Truncated granulin peptides. Biomolecules.

[19] Robinson S.D., Norton R.S. (2014). Conotoxin gene superfamilies. Mar. Drugs.

[20] Cruz L.J., Ramilo C.A., Corpuz G.P., Olivera B.M. (1992). Conus peptides: phylogenetic range of biological activity. Biol. Bull..

[21] Kaas Q., Westermann J.C., Halai R., Wang C.K., Craik D.J. (2008). ConoServer, a database for conopeptide sequences and structures. Bioinformatics.

[22] Kaas Q., Yu R., Jin A.H., Dutertre S., Craik D.J. (2012). ConoServer: updated content, knowledge, and discovery tools in the conopeptide database. Nucleic Acids Res..

[23] Klaus W., Broger C., Gerber P., Senn H. (1993). Determination of the disulphide bonding pattern in proteins by local and global analysis of nuclear magnetic resonance data. Application to flavoridin. J. Mol. Biol..

[24] Rosengren K.J., Daly N.L., Plan M.R., Waine C., Craik D.J. (2003). Twists, knots, and rings in proteins. Structural definition of the cyclotide framework. J. Biol. Chem..

[25] Daly N.L., Clark R.J., Craik D.J. (2003). Disulfide folding pathways of cystine knot proteins. Tying the knot within the circular backbone of the cyclotides. J. Biol. Chem..

[26] Ju S., Zhang Y., Guo X., Yan Q., Liu S., Ma B. (2022). Anti-ovarian Cancer conotoxins identified from Conus venom. Molecules.

[27] Postic G., Gracy J., Perin C., Chiche L., Gelly J.C. (2018). KNOTTIN: the database of inhibitor cystine knot scaffold after 10 years, toward a systematic structure modeling. Nucleic Acids Res..

[28] Le-Nguyen D., Heitz A., Chiche L., el Hajji M., Castro B. (1993). Characterization and 2D NMR study of the stable [9-21, 15-27] 2 disulfide intermediate in the folding of the 3 disulfide trypsin inhibitor EETI II. Protein Sci..

[29] Dutertre S., Jin A.H., Kaas Q., Jones A., Alewood P.F., Lewis R.J. (2013). Deep venomics reveals the mechanism for expanded peptide diversity in cone snail venom. Mol. Cell. Proteomics.

[30] Vranken W.F., Boucher W., Stevens T.J., Fogh R.H., Pajon A., Llinas M. (2005). The CCPN data model for NMR spectroscopy: development of a software pipeline. Proteins.

[31] Wuthrich K. (2003). NMR studies of structure and function of biological macromolecules (Nobel Lecture). J. Biomol. NMR.

[32] Shen Y., Bax A. (2015). Protein structural information derived from NMR chemical shift with the neural network program TALOS-N. Methods Mol. Biol..

[33] Guntert P. (2004). Automated NMR structure calculation with CYANA. Methods Mol. Biol..

[34] Koradi R., Billeter M., Wuthrich K. (1996). MOLMOL: a program for display and analysis of macromolecular structures. J. Mol. Graph..

[35] Berman H., Henrick K., Nakamura H. (2003). Announcing the worldwide protein data bank. Nat. Struct. Biol..

[36] Ulrich E.L., Akutsu H., Doreleijers J.F., Harano Y., Ioannidis Y.E., Lin J. (2008). BioMagResBank. Nucleic Acids Res..

[37] Tolkatchev D., Malik S., Vinogradova A., Wang P., Chen Z., Xu P. (2008). Structure dissection of human progranulin identifies well-folded granulin/epithelin modules with unique functional activities. Protein Sci..

[38] Wishart D.S., Bigam C.G., Holm A., Hodges R.S., Sykes B.D. (1995). 1H, 13C and 15N random coil NMR chemical shifts of the common amino acids. I. Investigations of nearest-neighbor effects. J. Biomol. NMR.

