# Supplementary material for: Structural analysis of a U-superfamily conotoxin containing a mini-granulin fold: Insights into key features that distinguish between the ICK and granulin folds
Source: J Biol Chem. 2024 Mar 18;300(4):107203. doi: 10.1016/j.jbc.2024.107203 (PMC11035057; doi:10.1016/j.jbc.2024.107203)
Supplement: Supporting Information [file mmc1.docx]

**Structural analysis of a U-superfamily conotoxin containing a mini-granulin fold: Insights into key features that distinguish between the ICK and granulin folds.**

**Tiziano Raffaelli^1^, David T. Wilson^1^, Sebastien Dutertre^2^, Julien Giribaldi^2#^, Irina Vetter^3, 4^, Samuel D Robinson^3^, Ashvriya Thapa^3, 4^, Antin Widi^1^, Alex Loukas^1^ and Norelle L. Daly^1*^**

^1^Australian Institute of Tropical Health and Medicine, James Cook University, Cairns, Australia; ^2^IBMM, Univ Montpellier, CNRS, ENSCM, 34095 Montpellier, France; ^#^current affiliation Genentech, 1 DNA Way, South San Francisco, CA, 94080, USA. ^3^Institute for Molecular Bioscience, The University of Queensland, QLD, 4072 Australia. ^4^ School of Pharmacy, The University of Queensland, QLD 4102, Australia.

^∗^Correspondence should be addressed to: Norelle L. Daly, Australian Institute of Tropical Health and Medicine, James Cook University, QLD 4878, Australia. Tel.: +61 [(07) 4232 1815](mailto:(07)%204232%201815), E-mail: [norelle.daly@jcu.edu.au](mailto:norelle.daly@jcu.edu.au)

Supporting Figures


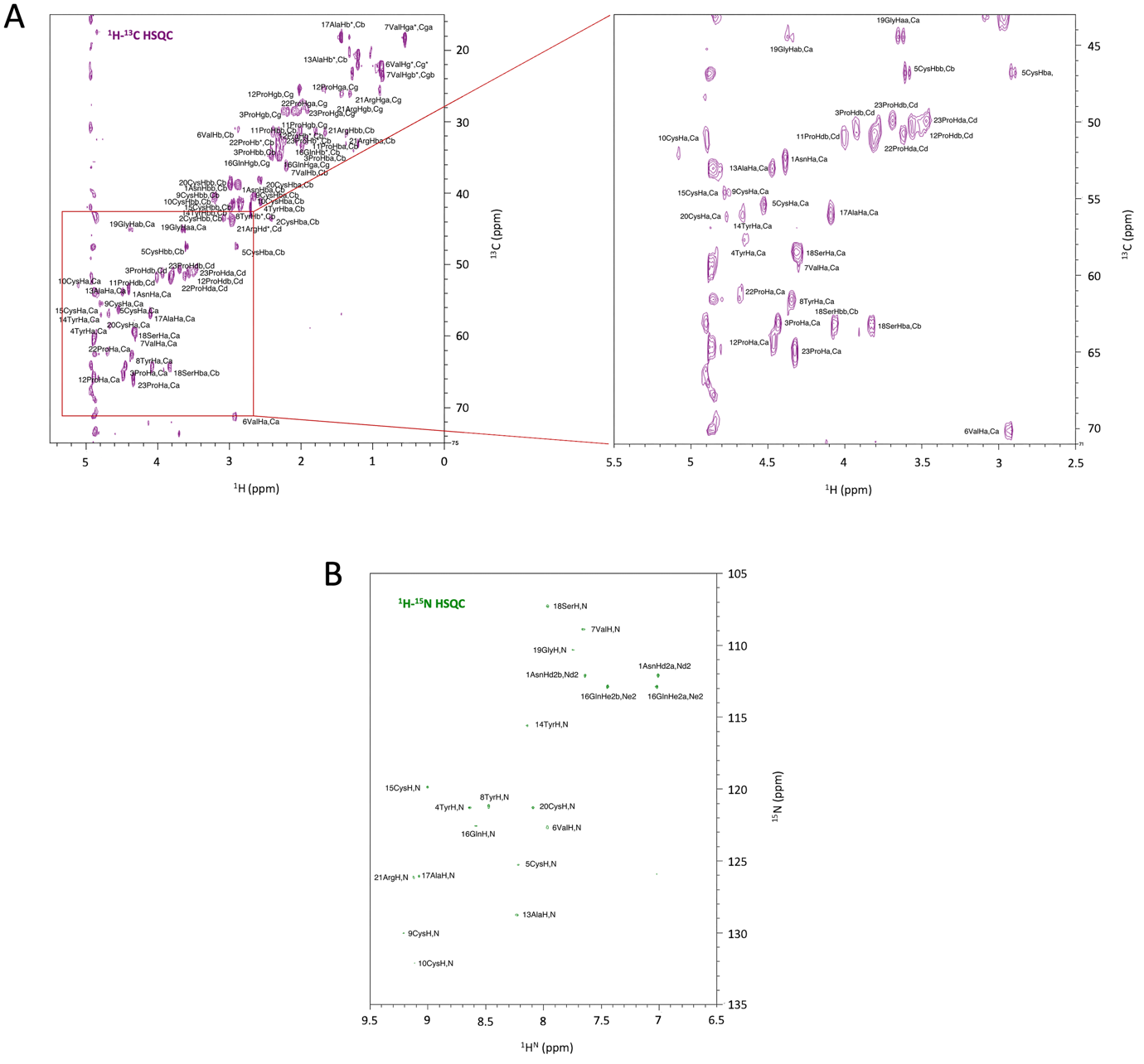


**Figure S1. HSQC NMR spectra of native TxVIIB peptide with resonance assignment.** Sample was dissolved in a solution of 500 μL water and 50 μL D_2_O, for a final peptide concentration of 0.2 mM. The experiments were carried out at 290 K using a 600 MHz Bruker Avance III spectrometer (see Experimental procedures). **(A)** [^1^H,^13^C]-HSQC spectrum of TxVIIB (left) and zoomed carbon alpha region (right); **(B)** [^1^H,^15^N]-HSQC spectrum of TxVIIB annotated with resonance assignment.


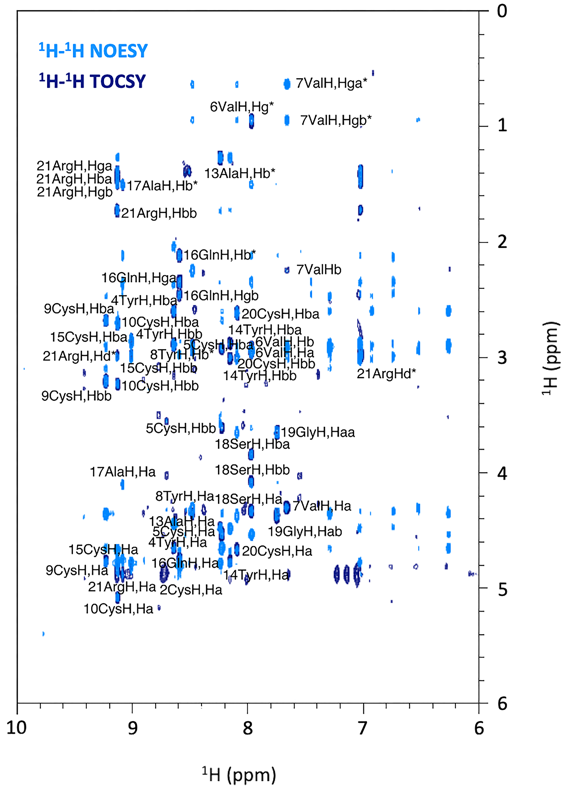


**Figure S.2 [^1^H,^1^H]-NOESY and [^1^H,^1^H]-TOCSY NMR spectra of native TxVIIB peptide.** Amide region of NOESY spectrum in light blue, and TOCSY spectrum with resonance assignment in navy. Sample was dissolved in a solution of 500 μL water and 50 μL D_2_O, for a final peptide concentration of 0.2 mM. The experiments were carried out at 290 K using a 600 MHz Bruker Avance III spectrometer (see Experimental procedures).


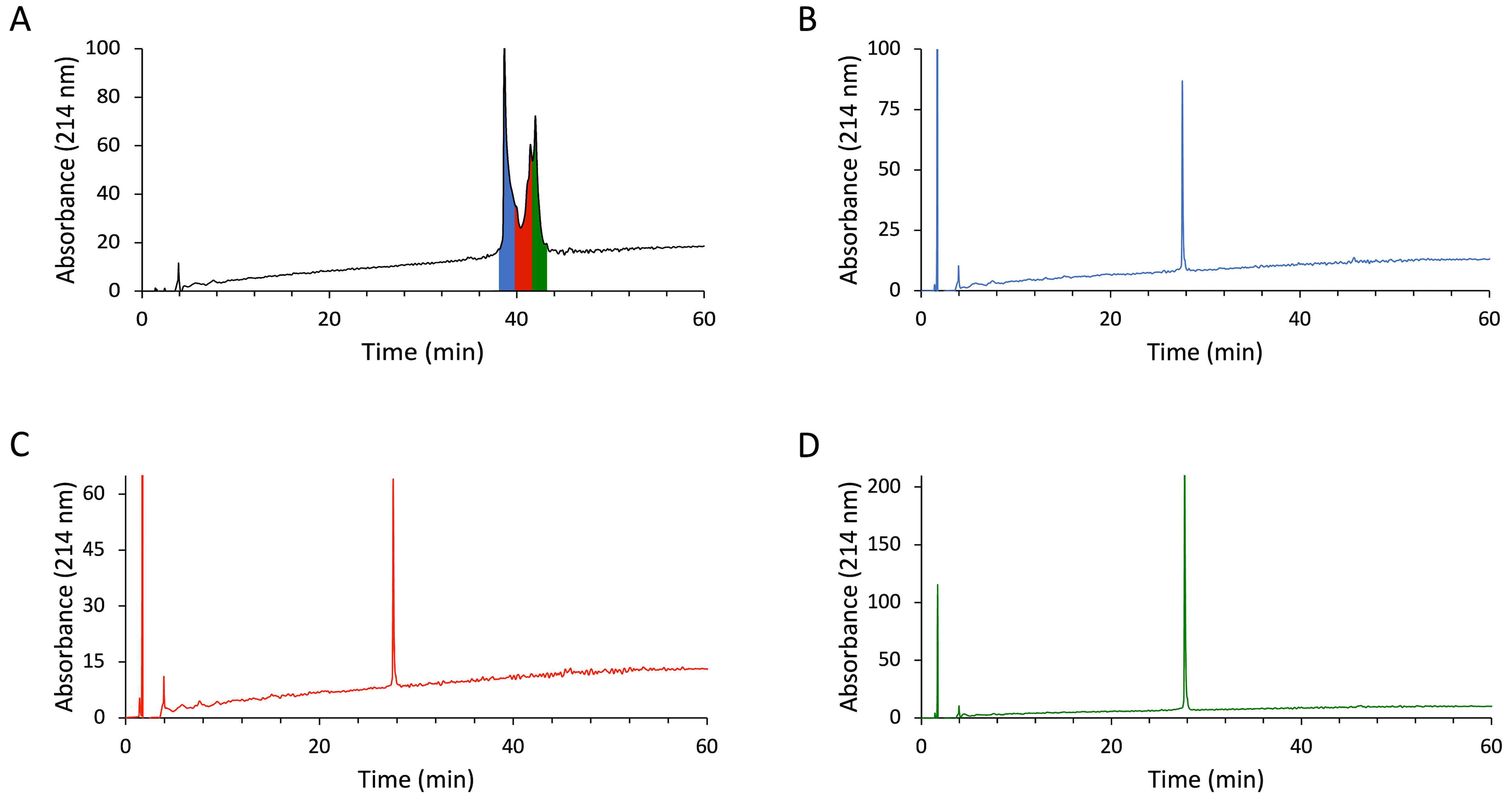


**Figure S3. Formation of the final disulfide bond in the selective protection of TxVIIB.** The two main peaks generated after oxidation II (blue and green respectively), and the fraction in between (in red), were collected individually and freeze-dried. Subsequently, a small amount of each fraction underwent independent oxidation to form the final disulfide bond. **(A)** Analytical RP-HPLC of purified TxVIIB with its first two disulfide bonds (Cys^5^-Cys^15^, Cys^9^-Cys^20^). The two peaks (blue and green) have the same mass but appear to be topological isomers. **(B)** Analytical RP-HPLC of the TxVIIB first peak after the third oxidation, with a retention time of 27.6 min. **(C)** Analytical RP-HPLC of the TxVIIB fraction between the two primary peaks, following the third oxidation, with a retention time of 27.6 min. **(D)** Analytical RP-HPLC of the TxVIIB second peak after the third oxidation, with a retention time of 27.7 min.


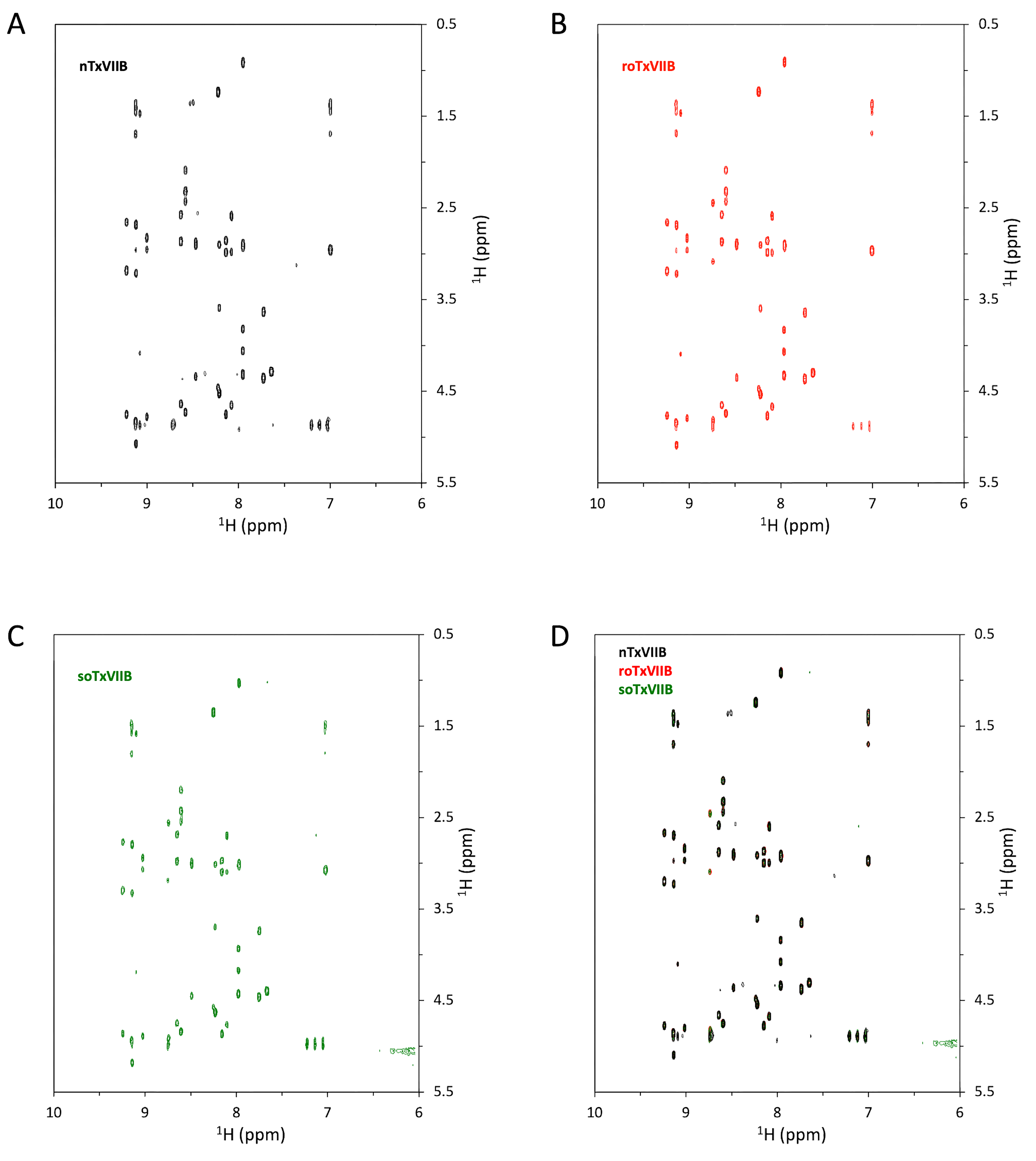


**Figure S4. Comparison of [^1^H,^1^H]-TOCSY NMR spectra of native and synthetic TxVIIB peptides.** Samples were dissolved in a solution of 500 μL water and 50 μL D_2_O, for a final peptide concentration of 0.2 mM. The experiments were carried out at 290 K using a 600 MHz Bruker Avance III spectrometer (see Experimental procedures). **(A)** Native TxVIIB; **(B)** Randomly oxidised TxVIIB; **(C)** Stepwise oxidised TxVIIB; and **(D)** spectrum superposition of all three TxVIIB peptides.


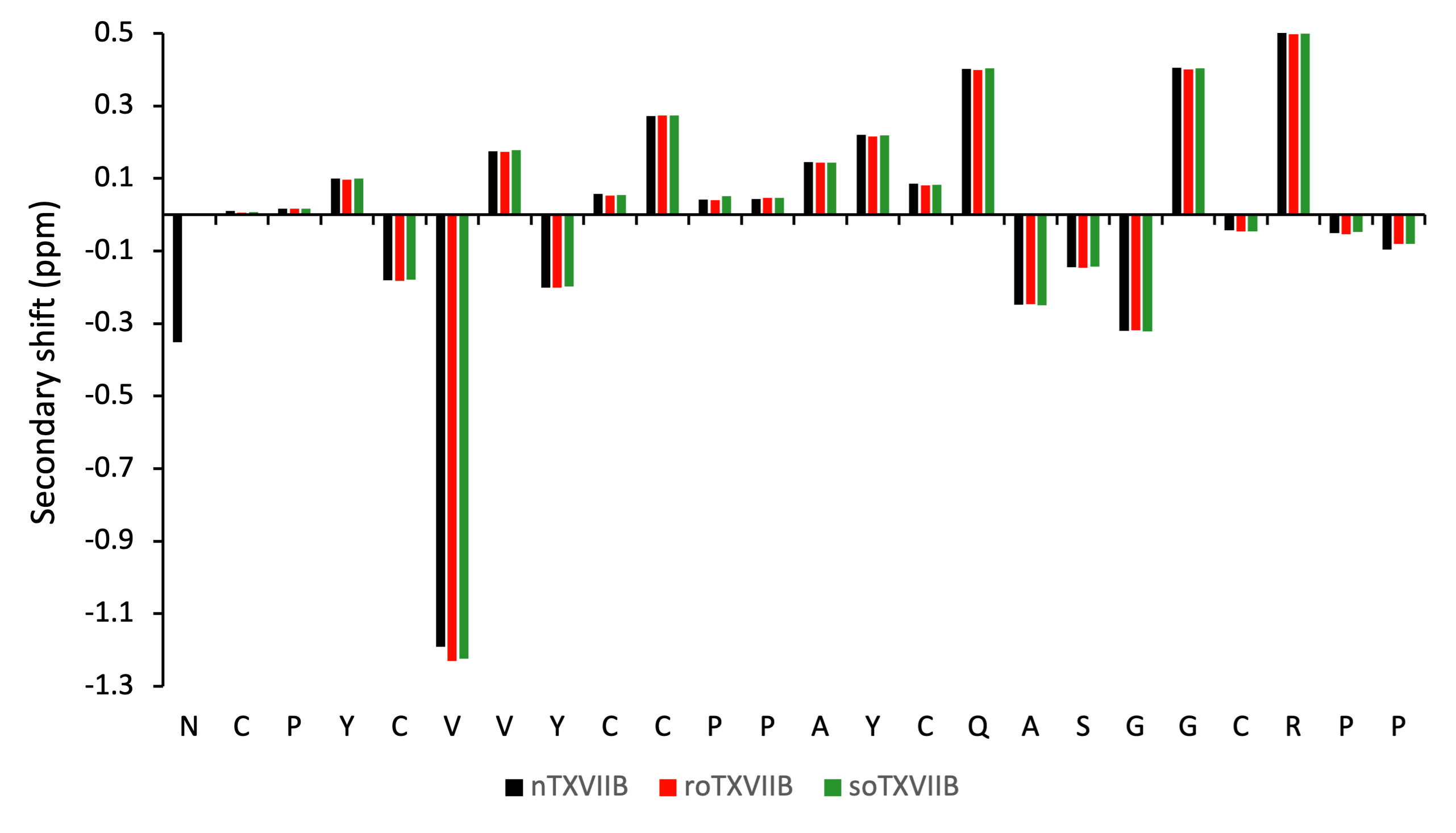


**Figure S5. Comparison of** αH secondary-shifts for nTxVIIB, roTxVIIB, and soTxVIIB. The αH secondary shifts were calculated by subtracting the random coil ^1^H NMR chemical shifts previously reported by Wishart et al. (1) from the experimental αH chemical shifts. The sequence of the peptides is given at the bottom of the diagram. The similarities in the chemical shifts indicate that the peptide have the same structures.


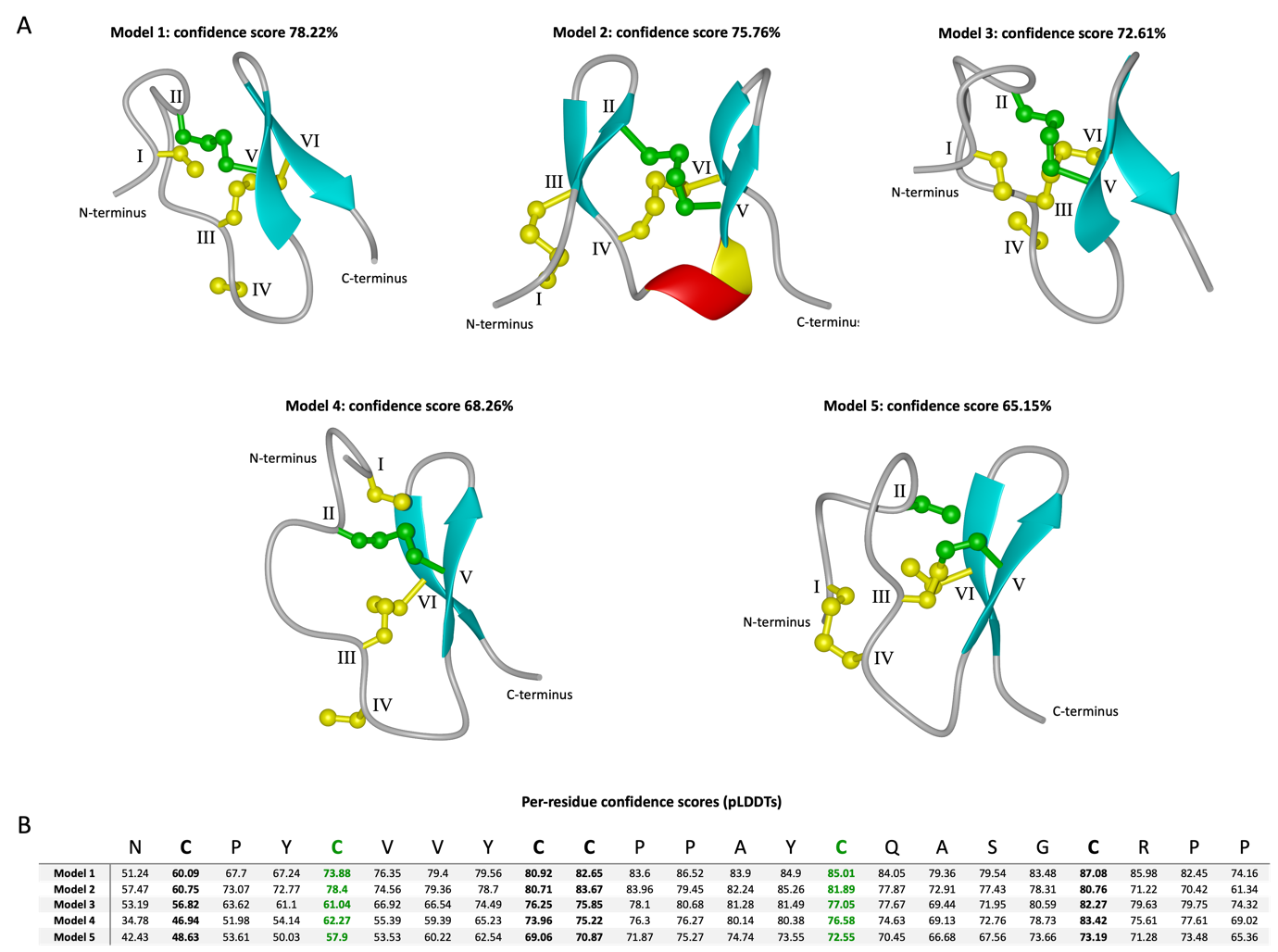


**Figure S6. Ensemble of the five AlphaFold predicted models for TxVIIB and per-residue confidence scores. (A) Three-dimensional structure and disulfide connectivity of the five AlphaFold predictions with the highest confidence scores.** The cysteine residues are labelled using Roman numerals (I-VI), while the β-sheets are coloured in cyan, and α-helix in red and yellow. The disulfide bond Cys^II^-Cys^V^ shared with TxVIIB is coloured in green; the other connectivities are in yellow. (B) Per-residue confidence scores table for the five predicted models. The table displays the pLDDTs (per-residues Local Distance Difference Test scores) for every residue of the five models. The cysteine residues forming the disulfide bond Cys^II^-Cys^V^ are highlighted in green, while the other cysteine residues are in bold.


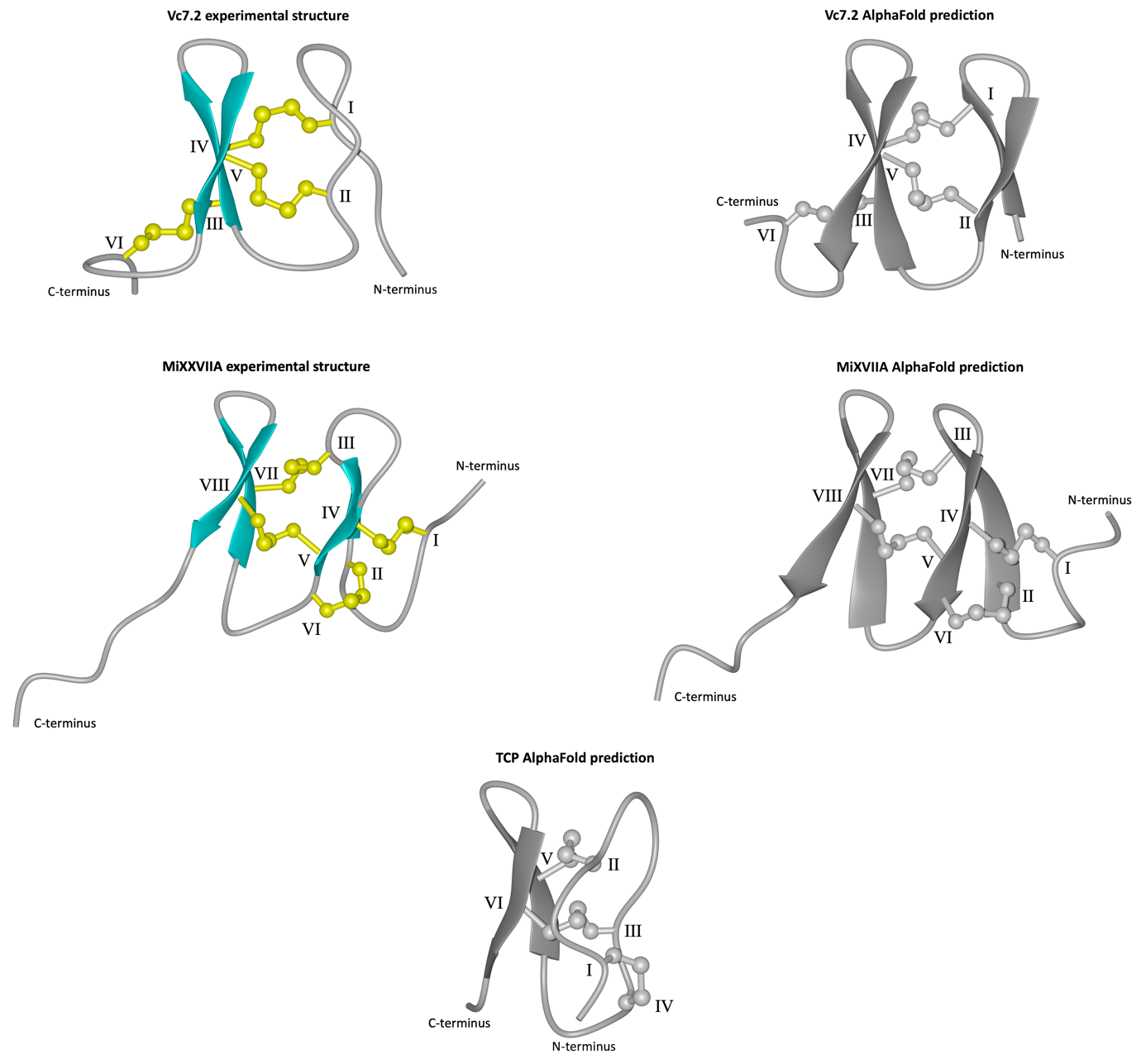


**Figure S7. Comparison of experimentally determined structures and AlphaFold prediction for H-Vc7.2,** Φ**-MiXVVIIA and TCP. The peptide structures experimentally calculated (**H-Vc7.2 PDB code 6Q5Z and Φ-MiXXVIIA PDB code 6PPC) **are represented in colour.** The cysteine residues are labelled using Roman numerals (I-VIII), the disulfide bonds are coloured in yellow, while the β-sheets in cyan. AlphaFold predictions of the three peptides are greyed-out, with cysteine residues labelled using Roman numerals (I-VIII). **The figure demonstrates a high degree of similarity between the experimentally determined and the predicted structures for H-Vc7.2 and** Φ-MiXXVIIA. TCP prediction displays an ICK disulfide connectivity in contrast to the granulin connectivity experimentally determined with mass spectrometry (2).

**References**

1. Wishart, D. S., Bigam, C. G., Holm, A., Hodges, R. S., and Sykes, B. D. (1995) 1H, 13C and 15N random coil NMR chemical shifts of the common amino acids. I. Investigations of nearest-neighbor effects. *J Biomol NMR* **5**, 67-81

2. Ju, S., Zhang, Y., Guo, X., Yan, Q., Liu, S., Ma, B., Zhang, M., Bao, J., Luo, S., and Fu, Y. (2022) Anti-Ovarian Cancer Conotoxins Identified from Conus Venom. *Molecules* **27**
